# Supplementary figures and images for: Hampered motility promotes the evolution of wrinkly phenotype in Bacillus subtilis
Source: BMC Evol Biol. 2018 Oct 16;18:155. doi: 10.1186/s12862-018-1266-2 (PMC6192195; doi:10.1186/s12862-018-1266-2)

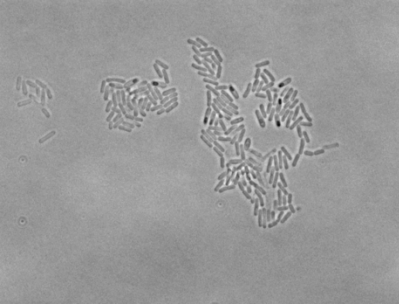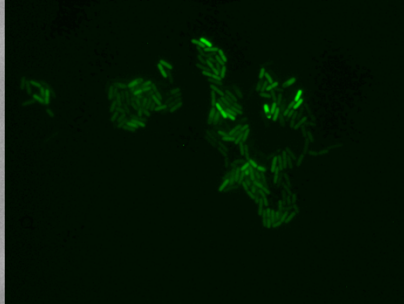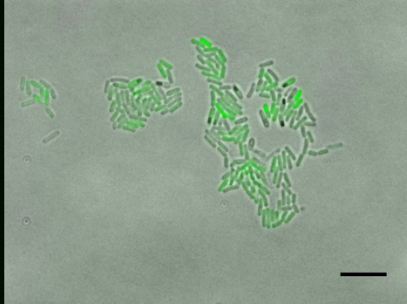

Supplement: Supplementary file 3 — Figure S1. Prolonged incubation of wild-type B. subtilis in LB medium results in aggregation with increased, but heterogeneous tapA expression. Representative microscopy images of strains harboring the PtapA-yfp reporter in wild type background. Images were recorded as in Fig. 2, but after longer (> 12 h) incubation in LB medium. The presented aggregates were observed in addition to homogeneously dispersed cells similar to those observed in Fig. 2. The scale bar represents 10 μm. (PDF 562 kb) [file 12862_2018_1266_MOESM2_ESM.pdf]

**WT**

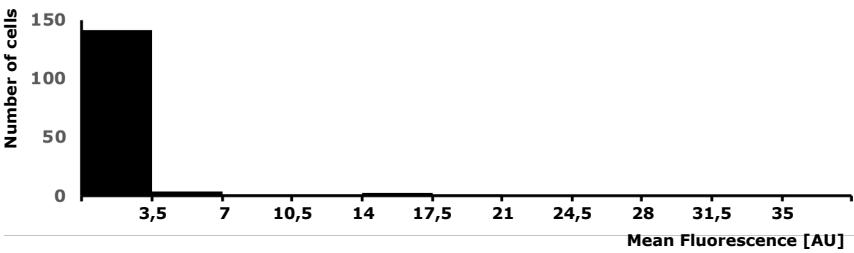

***Δhag***

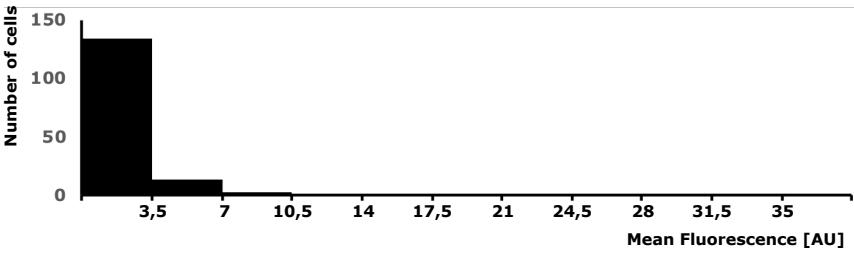

***ΔsinR***

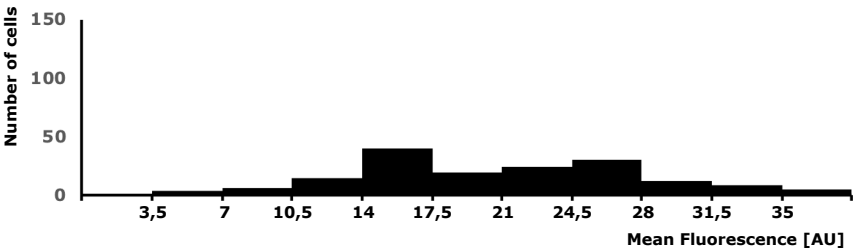

**WTWS1**

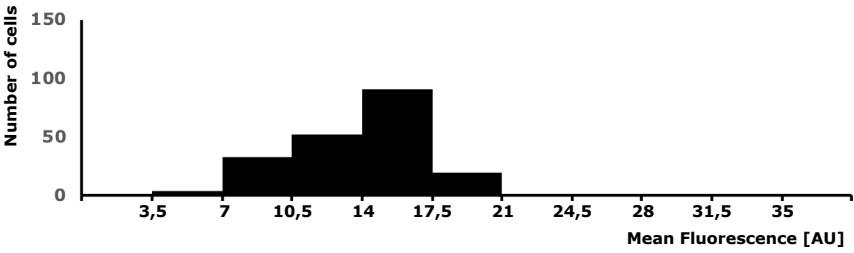

**WTWS8**

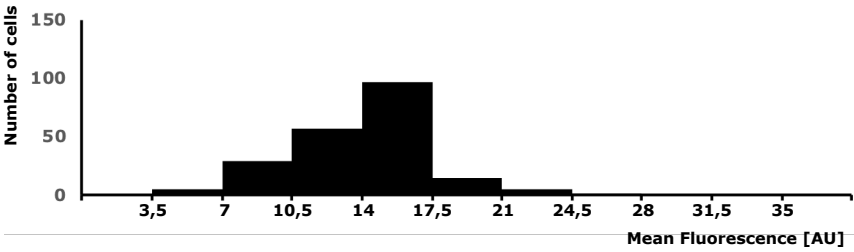

***Δhag*WS2**

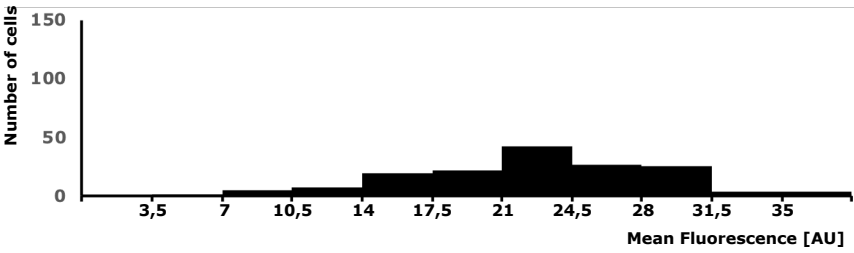

***Δhag*WS9**

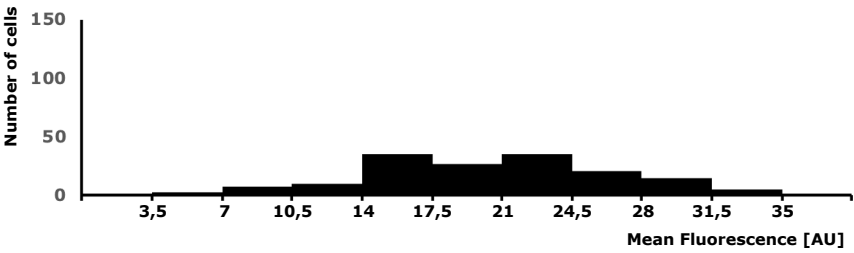

Supplement: Supplementary file 4 — Figure S2. Expression from the PtapA-yfp reporter detected at single cell level. Histograms were created based on images similar to Fig. 2a after randomly selecting 150 bacterial cells and detecting mean fluorescence using Image J (version 2.0.0-rc-68/1.52e). Background fluorescence was determined for each image based on 5 random selected positions where no cells were visible, and the average value was subtracted from each fluorescence data measured for the given image. X axis indicates mean fluorescence (arbitrary units), while Y axis denotes the number of cells. Raw data available in Additional file 1: Supplementary Dataset 1 (PDF 130 kb) [file 12862_2018_1266_MOESM3_ESM.pdf]

WT

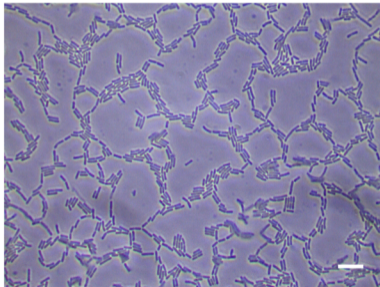

$P_{\text{hyperspank}}\text{-sinI}^{\text{full}}$

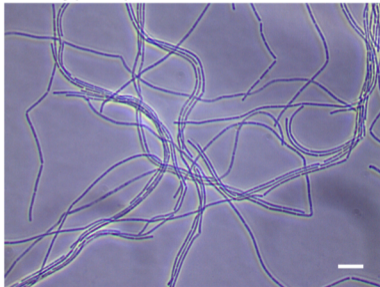

$P_{\text{hyperspank}}\text{-sinI}^{9-39}$

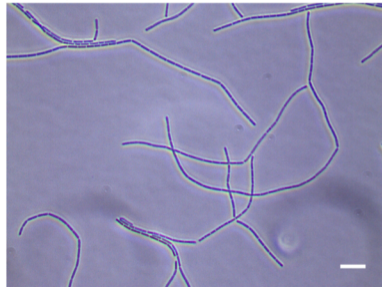

Supplement: Supplementary file 5 — Figure S3. Truncated SinI supports cell chaining. Microscopy images of wild type and two B. subtilis strains harboring a sinI overexpression construct of the full gene (sinIfull) or a truncated version (sinI9–39). The scale bar represents 10 μm. (PDF 1889 kb) [file 12862_2018_1266_MOESM4_ESM.pdf]

**a**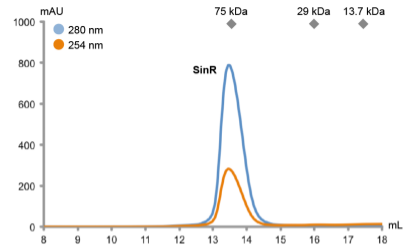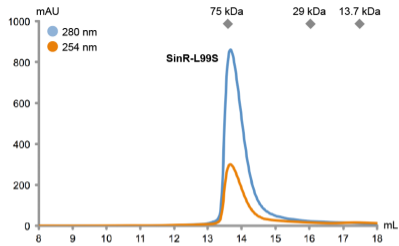**b**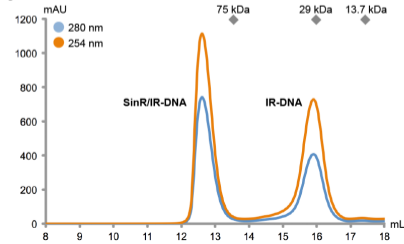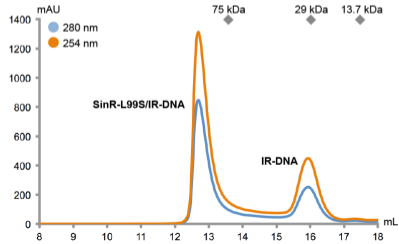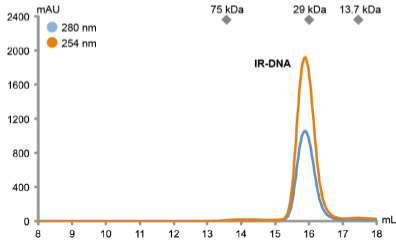

Supplement: Supplementary file 6 — Figure S4. SinR and SinR-L99S function as tetramers. (a) Analytical size exclusion chromatograms of SinR (left) and SinR-L99S (right). Runs were performed on a Superdex 200 Increase 10/300 GL column. The absorbance was recorded at 254 nm (red curve) and 280 nm (blue curve) in mAU (arbitrary units). (b) Analytical size exclusion chromatograms of the reconstituted SinR/IR-DNA complex (left), the SinR-L99S/IR-DNA complex (middle) and the individual IR-DNA duplex. Runs were performed on a Superdex 200 Increase 10/300 GL column. The absorbance was recorded at 254 nm (red curve) and 280 nm (blue curve) in mAU (arbitrary units). (PDF 255 kb) [file 12862_2018_1266_MOESM5_ESM.pdf]

dimerization  
interface

dimerization  
interface

SinR-A

SinR-B

N

C

C'

N'

5'

3'

5'

3'

inverted repeat DNA

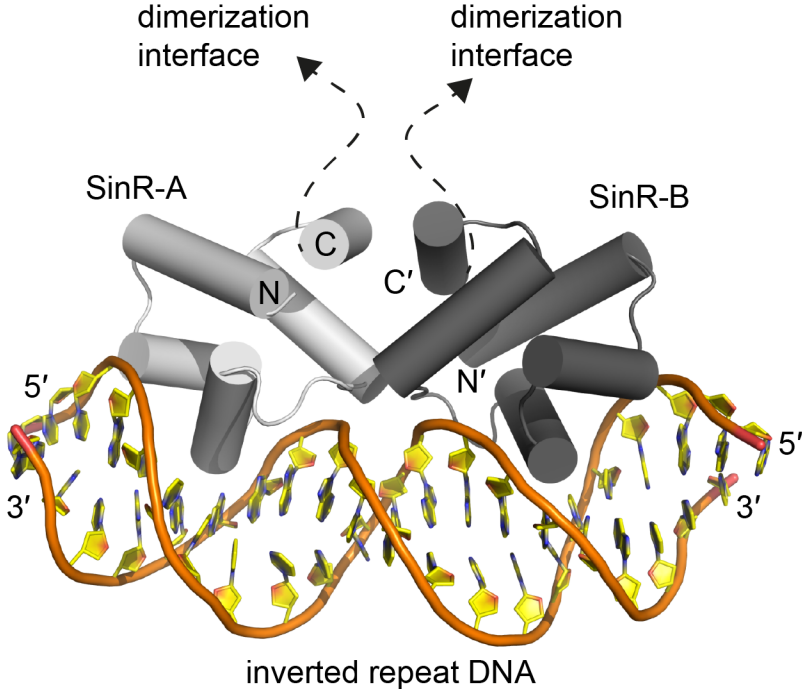

Supplement: Supplementary file 7 — Figure S5. Crystal structure of the SinR/DNA complex. The crystal structure of the B. subtilis SinR/IR-DNA complex is shown in cartoon representation (PDB-ID: 3ZKC; [23]). Two SinR proteins (SinR-A, colored in light grey and SinR-B, colored in dark grey) bind via their N-terminal DNA interaction domain to inverted repeat DNA. N and C indicate N-termini and C-termini, respectively. (PDF 592 kb) [file 12862_2018_1266_MOESM6_ESM.pdf]
