# Supplementary material for: Hampered motility promotes the evolution of wrinkly phenotype in Bacillus subtilis
Source: BMC Evol Biol. 2018 Oct 16;18:155. doi: 10.1186/s12862-018-1266-2 (PMC6192195; doi:10.1186/s12862-018-1266-2)
Supplement: Supplementary file 8 — Table S1. Strains and plasmids used in the current study. Strains labeled with *might contain additional mutations. (DOCX 23 kb) [file 12862_2018_1266_MOESM8_ESM.docx]

| Name | Genotype and features | Source |
| --- | --- | --- |
| *E. coli* |  |  |
| BL21 (DE3) | *fhuA*2 [*lon*] *ompT* *gal* (λDE3) [*dcm*] Δ*hsdS* | Novagen |
|  |  |  |
| *B. subtilis* |  |  |
| NCIB3610 | prototroph | [1] |
| DS1677 | NCIB3610 Δ*hag* | [2] |
| DS4681 | NCIB3610 Δ*flgE* | [3] |
| DS7080 | NCIB3610 Δ*fliF* | [4] |
| DS7498 | NCIB3610 Δ*motA* | [4] |
| DS6420 | NCIB3610 Δ*sigD* | [5] |
| DK1042 | NCIB3610 comI^Q12I^ | [6] |
| GP901 | Δ*hag*::Km^R^ | Jörg Stülke, lab collection |
| TB406 | NCIB3610 comI^Q12I^ Δ*sinR*::Cm | this work |
| 168 hyGFP | 168 *amyE*::P_hyperspank_-GFP; Cm^R^ | [7] |
| 168 hymKATE2 | 168 *amyE*::P_hyperspank_-mKATE2; Cm^R^ | [7] |
| TB34 | NCIB3610 *comI*^Q12I^ *amyE*::P_hyperspank_-GFP; Cm^R^ | [8] |
| TB35 | NCIB3610 *comI*^Q12I^ amyE::P_hyperspank_-mKATE2; Cm^R^ | [8] |
| WTWS1* | *comI*^Q12I^ *sinR*^L99S^ | this work |
| WTWS4* | *comI*^Q12I^ *sinR*^A85T^ | this work |
| WTWS8* | *comI*^Q12I^ *sinR*^V26G^ | this work |
| WTWS9* | *comI*^Q12I^ *sinR*^V26G^ | this work |
| Δ*hag*WS1* | *comI*^Q12I^ Δ*hag*::Km^R^ *sinR*^Q108stop^ | this work |
| Δ*hag*WS2* | *comI*^Q12I^ Δ*hag*::Km^R^ *sinR*^L99S^ | this work |
| Δ*hag*WS9* | *comI*^Q12I^ Δ*hag*::Km^R^ *sinR*^V26G^ | this work |
| TB773 | WTWS1 *comI*^Q12I^ *sinR*^L99S^ *amyE*::P_hyperspank_-GFP; Cm^R^ | this work |
| TB774 | WTWS1 *comI*^Q12I^ *sinR*^L99S^ *amyE*::P_hyperspank_-mKATE2; Cm^R^ | this work |
| TB282 | NCIB3610 *comI*^Q12I^ Δ*hag*::Km^R^ | this work |
| TB36 | NCIB3610 *comI*^Q12I^ Δ*hag*::Km^R^ *amyE*::P_hyperspank_-GFP; Cm^R^ | this work |
| TB37 | NCIB3610 *comI*^Q12I^ Δ*hag*::Km^R^ *amyE*::P_hyperspank_-mKATE2; Cm^R^ | this work |
| TB775 | Δ*hag*WS2 *comI*^Q12I^ Δ*hag*::Km^R^ *sinR*^L99S^ *amyE*::P_hyperspank_-GFP; Cm^R^ | this work |
| TB776 | Δ*hag*WS2 *comI*^Q12I^ Δ*hag*::Km^R^ *sinR*^L99S^ *amyE*::P_hyperspank_-mKATE2; Cm^R^ | this work |
| TB697 | NCIB3610 *comI*^Q12I^ *amyE*::P_hyperspank_-*sinI* *lacI*; Spec^R^ | this work |
| TB698 | NCIB3610 *comI*^Q12I^ *amyE*::P_hyperspank_-*sinI*^9-39^ *lacI*; Spec^R^ | this work |
| DL821 | NCIB3610 *lacA*::P*_tapA_*-*yfp*; MLS^R^ | [9] |
| TB699 | NCIB3610 *comI*^Q12I^ *lacA*::P*_tapA_*-*yfp*; MLS^R^ | this work |
| TB778 | NCIB3610 *comI*^Q12I^ Δ*hag*; *lacA*::P*_tapA_*-*yfp*; MLS^R^ | this work |
| TB777 | NCIB3610 *comI*^Q12I^ Δ*sinR*; *lacA*::P*_tapA_*-*yfp*; MLS^R^ | this work |
| TB700 | WTWS1 *comI*^Q12I^ *sinR*^L99S^ *lacA*::P*_tapA_*-*yfp*; MLS^R^ | this work |
| TB701 | WTWS8 *comI*^Q12I^ *sinR*^V26G^ *lacA*::P*_tapA_*-*yfp*; MLS^R^ | this work |
| TB702 | Δ*hag*WS2 *comI*^Q12I^ Δ*hag*::Km^R^ *sinR*^L99S^ *lacA*::P*_tapA_*-*yfp*; MLS^R^ | this work |
| TB703 | Δ*hag*WS9 *comI*^Q12I^ Δ*hag*::Km^R^ *sinR*^V26G^ *lacA*::P*_tapA_*-*yfp*; MLS^R^ | this work |
|  |  |  |
| plasmids |  |  |
| pDR111 | *amyE* integration vector; P_hyperspank_; *lacI*; Amp^R^, Spec^R^ | David Rudner, lab collection |
| pTB695 | *sinI* cloned into pDR111; Amp^R^, Spec^R^ | this work |
| pTB696 | *sinI*^9-39^ cloned into pDR111; Amp^R^, Spec^R^ | this work |
| pET24d | IPTG inducible overexpression vector; Km^R^ | Novagen |
| pET24sinR^WT^ | wild type *sinR* cloned into pET24d; Km^R^ | this work |
| pET24sinR^V26G^ | *sinR*^V26G^ cloned into pET24d; Km^R^ | this work |
| pET24sinR^A85D^ | *sinR*^A85T^ cloned into pET24d; Km^R^ | this work |
| pET24sinR^L99S^ | *sinR*^L99S^ cloned into pET24d; Km^R^ | this work |

**References**

1. Branda SS, González-Pastor JE, Ben-Yehuda S, Losick R, Kolter R. Fruiting body formation by *Bacillus subtilis*. Proc Natl Acad Sci U S A. 2001;98:11621–6.

2. Blair KM, Turner L, Winkelman JT, Berg HC, Kearns DB. A molecular clutch disables flagella in the *Bacillus subtilis* biofilm. Science. 2008;320:1636–8.

3. Courtney CR, Cozy LM, Kearns DB. Molecular characterization of the flagellar hook in *Bacillus subtilis*. J Bacteriol. 2012;194:4619–29.

4. Chan JM, Guttenplan SB, Kearns DB. Defects in the flagellar motor increase synthesis of poly-γ-glutamate in *Bacillus subtilis*. J Bacteriol. 2014;196:740–53.

5. Cozy LM, Phillips AM, Calvo RA, Bate AR, Hsueh YH, Bonneau R, et al. SlrA/SinR/SlrR inhibits motility gene expression upstream of a hypersensitive and hysteretic switch at the level of σD in *Bacillus subtilis*. Mol Microbiol. 2012;83:1210–28.

6. Konkol MA, Blair KM, Kearns DB. Plasmid-encoded comI inhibits competence in the ancestral 3610 strain of *Bacillus subtilis*. J Bacteriol. 2013;195:4085–93.

7. van Gestel J, Weissing FJ, Kuipers OP, Kovács ÁT. Density of founder cells affects spatial pattern formation and cooperation in *Bacillus subtilis* biofilms. ISME J. 2014;8:2069–79.

8. Hölscher T, Dragoš A, Gallegos-Monterrosa R, Martin M, Mhatre E, Richter A, et al. Monitoring spatial segregation in surface colonizing microbial populations. J Vis Exp. 2016;2016:e54752.

9. López D, Vlamakis H, Losick R, Kolter R. Paracrine signaling in a bacterium. Genes Dev. 2009;23:1631–8.
