# Supplementary material for: Hampered motility promotes the evolution of wrinkly phenotype in Bacillus subtilis
Source: BMC Evol Biol. 2018 Oct 16;18:155. doi: 10.1186/s12862-018-1266-2 (PMC6192195; doi:10.1186/s12862-018-1266-2)
Supplement: Supplementary file 9 — Table S2. Oligonucleotides used in the current study. (DOCX 12 kb) [file 12862_2018_1266_MOESM9_ESM.docx]

| Name | Sequence | Feature |
| --- | --- | --- |
| oTB98 | GGCCGTCTCGATGGTTATTG | *sinIR* locus sequencing |
| oTB99 | GGCCGGACTGGCTGAAATAC | *sinIR* locus sequencing |
| oTB124 | CTGAAGCTTAGGAGGAGAAACTGCATGAAG | *sinI* cloning |
| oTB125 | CATGGCATGCGCACATTCAGAAAGGATTTAC | *sinI* cloning |
| oTB126 | CTGAAGCTTAGGAGGAGAAACTGCATGTTTGAATTGGATCAAGAATGG | *sinI* cloning  (shortened) |
| oTB127 | CATGGCATGCGCACATTCAGTTTAAAAGTAAATATTTTCGTATTTC | *sinI* cloning  (shortened) |
| SinR_NcoI_F | TATACCATGGGCATTGGCCAGCGTATTAAAC | *sinR* overexpression |
| SinR_H6_BamHI_R | TAATGGATCCTTAGTGATGGTGATGGTGATGCTCCTCTTTTTGGGATTTTCTCC | *sinR* overexpression |
| SinR_V26G_F | GAAAAAGCTGGGGGCGCGAAGTCTTA | V26G mutagenesis |
| SinR_ V26G _R | TAAGACTTCGCGCCCCCAGCTTTTTC | V26G mutagenesis |
| SinR_A85D_F | GGTTCGCGATGATATGACATCCGG | A85D mutagenesis |
| SinR_ A85D _R | CCGGATGTCATATCATCGCGAACC | A85D mutagenesis |
| SinR_L99S_F | CGTGAATTTAGCGATTATCAAAAATG | L99S mutagenesis |
| SinR_ L99S_R | CATTTTTGATAATCGCTAAATTCACG | L99S mutagenesis |
| SinR_IR_F | TTTGTTCTCTAAAGAGAACTTA | SinR binding site |
| SinR_IR_R | TAAGTTCTCTTTAGAGAACAAA | SinR binding site |
